# The Effect of Soil on the Biochemical Plasticity of Berry Skin in Two Italian Grapevine (*V. vinifera* L.) Cultivars

**DOI:** 10.3389/fpls.2020.00822

**Published:** 2020-06-26

**Authors:** Corrado Perin, Aaron Fait, Fabio Palumbo, Margherita Lucchin, Alessandro Vannozzi

**Affiliations:** ^1^ Department of Agronomy, Food, Natural Resources, Animals and Environment, University of Padova, Legnaro PD, Italy; ^2^ The French Associates Institute for Agriculture and Biotechnology of Drylands, the Jacob Blaustein Institute for Desert Research, Ben-Gurion University of the Negev, Sede Boqer, Israel

**Keywords:** Corvina, Glera, Genotype-per-Environment interaction (G×E), LC-MS, phenotypic plasticity, secondary metabolism, terroir

## Abstract

Grapevine represents a particularly interesting species as concerns phenotypic plasticity, considering that the *terroir*, meaning the contribution of the geography, geology, and climate of a certain place, together with the agronomical practices utilized, may deeply influence the berry phenotype at the physiological, molecular, and biochemical levels. This phenomenon leads to the production of wines that, although produced from the same variety, exhibit different enological profiles and represents an issue of increasing interest from both a biological and an economic point of view. The main objective of the present study was to deepen the understanding of phenotypic plasticity in grapevine, trying to dissect the role of one its important components – the soil – by investigating the singular effect that different physico-chemical soil properties can produce in terms of berry plasticity at the phenological, physiological, and biochemical levels in a red and a white variety of great economic importance in Italy and overseas: Corvina and Glera. The results indicated a genotype-dependent response to the soil factor, with higher biochemical plasticity in Corvina with respect to Glera and suggested a key role of specific soil properties, including the skeleton, texture, and mineral composition, on the metabolite profile of berry skin.

## Introduction

In viticulture, the term terroir is generally used to link the typicity of a wine with its area of production (Fernández-Marín et al., 2013; Foroni et al., 2017). It refers to the specific signature that the combination of climate, soil, and agronomical practices confers to the quality of grapes and ultimately wine (Pereira et al., 2005; Roullier-Gall et al., 2014; Fabres et al., 2017; Foroni et al., 2017). From a genetic point of view, this term rather refers to the complex network of interactions that takes place between a given genotype and the surrounding environment and that manifests itself in a particular phenotype (Van Leeuwen and Seguin, 2006; Gladstones, 2011; Roullier-Gall et al., 2014; Fabres et al., 2017). The ability of a single genotype to give rise to alternative phenotypes when exposed to different environmental conditions is defined as phenotypic plasticity (Sultan, 2000). Grapevine berries are characterized by remarkable phenotypic plasticity, with a single clone showing variability for many traits at the level of single berry, between different berries on the same bunch, between multiple bunches on the same plant, and between different plants in a vineyard (Keller, 2010). This characteristic can be considered a burden, since berries may mature unevenly and display large inter-seasonal fluctuations in quality, but, at the same time, it offers several advantages, including the ability of a given genotype to adapt to specific growing regions and the possibility of obtaining different wines from the same variety (Dai et al., 2011). Among the main terroir factors (i.e., cultivar, soil, climate, agronomical practices) affecting phenotypic plasticity, soil is known to play a considerable role, contributing to the uniqueness of berry composition (Cheng et al., 2014; Zerihun et al., 2015) and the organoleptic properties of wine (Van Leeuwen and Seguin, 2006; Foroni et al., 2017). Unlike other terroir factors, such as climate, it is characterized by a great spatial variability, and it is not unusual to come across different types of soil even in narrow field parcels (Pereira et al., 2005). Although this high in-field variability makes it difficult to relate vine behavior to geology or geomorphology, in some regions, a correspondence between the type of geological outcrop and the typicity of the wine was observed (Van Leeuwen, 2010). Recently, some authors highlighted the role of soil in determining the final perceived quality of wine (Coipel et al., 2006; Robinson et al., 2012; Fabres et al., 2017), indicating it as a key factor for explaining differences in the quality of wines produced within the same geographical and climatic area (de Andrés-de Prado et al., 2007; Herderich et al., 2015).

The quality of grapevine berries mainly depends on the metabolites they accumulate, the biosynthesis of which is known to be sensitive to external conditions (Zhang et al., 2005). In particular, chemical diversity is mostly affected by secondary metabolites that play a very important role in the human taste perception, although present at low concentrations (Roullier-Gall et al., 2014). Grape secondary metabolites are predominantly phenylpropanoids, which are typically found in the berry skin and comprise flavonoids, phenolic acids, stilbenes, and viniferins (Anesi et al., 2015). The plasticity of phenylpropanoid metabolism is a well-known feature of grape berries and confers most of the wine quality characteristics that are associated with specific terroirs (Teixeira et al., 2013). The chemical composition of grapes and wine has been intensely studied in recent decades, and the number of compounds identified has increased exponentially since the development of different analytical techniques such as gas and liquid chromatography coupled with mass spectrometry (GC- and LC-MS). Despite its importance, the effect of soil and its intrinsic characteristics, such as texture, depth, chemical composition, fertility, and water availability, on grape and wine biochemical properties has not been deeply investigated so far (Seguin, 1986; Van Leeuwen et al., 2004). This is mainly due to its great spatial variability, even within the same field, and to the difficulties in isolating this factor from others in open field experiments. The only available information on the biochemical plasticity of berries is derived from studies that considered plants grown in different sites and were thus also exposed to other terroir variables, making it difficult to isolate the single effect of each one (Anesi et al., 2015; Dal Santo et al., 2016).

In the present study, we tried to dissect the singular effect of soil on berry development and biochemistry. A white and a red cultivar (Glera and Corvina, respectively) were grown on three different soils collected from three different areas of the Veneto region (Italy) but set in the same place, hence under the same climatic and agronomic conditions. These varieties were chosen because they are strictly linked to the territory for the typicity of their derived wines, Prosecco and Amarone, respectively, hence representing a perfect model for studying the molecular mechanisms linked to the terroir. Since secondary metabolism is considered to be central to shaping the sensory profile of grapes and wine, is highly sensitive to external conditions (Zhang et al., 2005), and is mainly related to grape exocarp, we performed biochemical analysis of berry skins by using LC-MS technology. We aimed to isolate the effect of different soils on the accumulation of secondary metabolites, to track correlations between metabolites and soil characteristics, and, ultimately, to identify the main physical and chemical parameters that contribute to the metabolic plasticity of berry skin.

Our study is meant as a first step in the study of the single effect of the soil factor on grape quality, revealing its potential importance on the final product, its contribution to the complexity of terroir, and proving the delicate balance existing between a variety and its typical area of production.

## Materials and Methods

### Experimental Design

**Figure 1:**
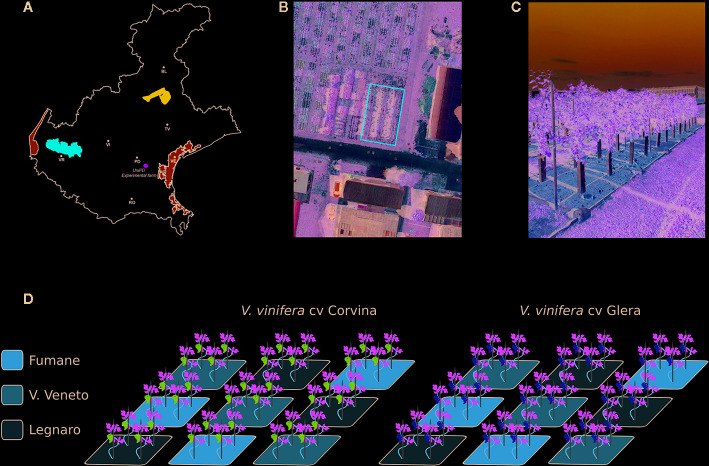
Experimental design. **(A)** Schematic map of the Veneto region indicating the three localities where soils were collected: the locality of Fumane (F) within the Valpolicella DOCG area (highlighted in red), the locality of Vittorio Veneto (VV) within the Prosecco Valdobbiadene-Conegliano area (highlighted in blue), and the L. Toniolo experimental farm of the University of Padua located in Legnaro (L) municipality (highlighted in green), where the experiment took place. **(B)** Satellite image showing the arrangement of the concrete caissons at the L. Toniolo experimental farm. **(C)** Picture showing the experimental plan for the 2017 vintage. **(D)** Schematic representation of the experimental plan: three soil boxes per thesis (F, VV, L), four plants per box, two varieties (Glera and Corvina). All plants were grafted onto the same rootstock (Kober 5BB).

The experiment was conducted during the 2018 growing season in a tailored experimental vineyard located at the L. Toniolo experimental farm belonging to the University of Padua (Legnaro, Italy) in the north-east of Italy. The area is characterized by a humid subtropical climate (www.it.climate-data.org) with hot and humid summers and cool to mild winters. In 2016, two-year-old certified clonal varieties of *V. vinifera* cv. Glera and Corvina grafted on the Kober 5BB rootstock genotype were transplanted into cement boxes (2 m x 2 m x 1.5 m depth) filled with three soils collected from different areas of the Veneto region linked to internationally renowned wine production. The first soil, indicated as “F,” was collected from a site located within the Fumane municipality (VR) (Lat. 45.54, Lon. 10.94, Alt. 244 m a.s.l.), part of the Valpolicella DOCG area and mainly recognized for Amarone winemaking. The second soil, designated “VV,” was collected from the hilly area of Vittorio Veneto (TV) (Lat. 45.95, Lon. 12.33, Alt. 95 m a.s.l.), part of the Prosecco DOCG area and known for the production of the homonymous wine. Finally, the third soil, designated as “L” and considered as an out layer, was represented by the low-plains soil of the L. Toniolo experimental farm (University of Padova, Legnaro PD) where the experiment took place (Lat. 45.35, Lon. 11.95, Alt. 8 m a.s.l.). Approximately 40 m^3^ of each soil was collected and transported to the site of the experiment by truck. To avoid collecting the deepest layers of soil, we limited ourselves to the first 30 cm of depth. The choice of harvest areas was based in part on the availability of the landowners, as well as on the homogeneity of the substrate. Due to the impossibility of maintaining the soil structure, given the size of the caissons and the presence of a skeleton in the soils of V. Veneto and Fumane, the soil of the experimental farm (L) was also mixed to make the samples comparable. The caissons containing the different soils had no base to avoid water stagnation. The choice of the Kober 5BB rootstock relies on its performance, which is quite stable and uniform under many different conditions and so does not facilitate the growth of plants in one substrate rather than in another. Each cultivar/soil combination was set in three replicates constituted of three independent boxes, each one containing four plants, for a total number of 18 boxes randomly distributed (2 cultivars x 3 soils x 3 biological replicates) and 72 plants (**Figure 1**). Plants were grown using a spurred cordon training system; the plant-to-plant distance was equal to 100 cm, and the rows were oriented north–south. No fertilization was carried out so as to not overshadow any possible difference in plant phenotype due to differences in soil chemical composition. To avoid introducing an additional variable, no irrigation was provided, considering the ability to retain water as an intrinsic feature of the different soils. The weeding was carried out mechanically and manually, while treatments with fungicides or pesticides were carried out following the regional protocols against grapevine leafhopper (*Scaphoideus titanus*) Cicadellidae) and against the development of powdery mildew (*Erysiphe necator*) and downy mildew (*Plasmopara viticola*).

### Soil Physico-Chemical Analyses

Soils were sampled by collecting a 20 cm-depth vertical core from the center of each box (six replicates per soil). Analyses were conducted according to the International Union of Soil Science (IUSS) protocols. A detailed description of protocols used for soil analyses is reported in **Supplementary Methods S1**.

### Canopy and Berry Physiological Analyses

From bud breaking to flowering, the internode growth and the leaf number of one representative shoot per plant were measured weekly as biometric indicators for assessing the vegetative growth. Later on, the effective leaf area index (LAIe) parameter was used as a canopy growth indicator, which was calculated weekly by processing the digital photos taken from underneath the canopy of each plant by using a free-download app developed by the School of Agriculture, Food, and Wine (University of Adelaide, Australia) (De Bei et al., 2016). The total soluble solids (TSS) content in °Brix was measured using a hand refractometer (Palette PR-100, Atago USA). These parameters were also used to determine the phenological stage according to the modified E-L system (Coombe, 1995). Measurements were performed on each singular plant (4 berries per plant) at three time points corresponding to complete véraison (‘intermediate Brix value' - 36 E-L), mid-ripening (‘berries not quite ripe' - 37 E-L), and harvest ripe (‘berries harvest-ripe' - 38 E-L) according to the phenological scale of the modified E-L system (Coombe, 1995). Conversely, titratable acidity (TA; g/L tartaric acid equivalents) and pH were measured on a pool of berries collected from the four plants belonging to each biological replicate (box), according to the standard procedures used in Guymon and Ough (1962). The maturation index (MI) was calculated as the TSS/TA ratio. At harvest, berry weight was averaged from at least 15 berries selected from the middle part of a representative bunch of each plant.

### Biochemical Analyses of Berry Skin

For biochemical analyses, berries were collected at two time points throughout the ripening phase, corresponding to E-L stages 36 and 38. Three berries were collected at the same time of the day (around 11 a.m.) from the middle external part of a representative cluster of each plant, avoiding those with visible damage or signs of pathogen infection and directly exposed to the sun. Berries from plants grown on the same soil box were pooled together to represent a single biological replicate (therefore constituted by 12 berries) then immediately frozen in liquid nitrogen. Skin was carefully separated from pulp and seeds by partially defrosting the outer berry layer with a sterilized cloth, peeling off the skin and freezing it again in liquid nitrogen. The dissection of skin and pulp from semi-frozen berries made the process much effective compared to using fresh berries and was based on previous studies (Fasoli et al., 2012). Skin samples were then manually ground by pestle and mortar under liquid nitrogen. For the LC-MS analysis, around 200 mg of ground sample was weighted and lyophilized. Metabolites were extracted following a protocol described in Weckwerth et al. (2004) with some adaptation to berry skin as detailed in Degu et al. (2014)(**Supplementary Methods S2**). Samples were run in an Ultra Performance Liquid Chromatography coupled with Quadrupole Time-of-Flight Mass-Spectrometry (UPLC-QTOF MS, Waters, MA, USA) system operating in positive and negative ion modes. LC-MS conditions were the same as described in Hochberg et al. (2013) and recently reviewed by Reshef et al. (2019). MassLynxTM software 4.1 (Waters^®^) was used as the system controlling the UPLC and for data acquisition. The raw data acquired were processed using MarkerLynx application manager (Waters Corporation, Milford, USA), as described in Hochberg et al. (2013). Metabolites were also annotated based on the consistency of their retention times with those of identified metabolites (Degu et al., 2014) and their fragmentation patterns crossed with the ChemSpider metabolites database (http://www.chemspider.com/).

### Data Normalization and Statistical Analyses

The ion counts of each detected metabolite were normalized to the internal standards (corticosterone, ampicillin) and the total ion count (TIC), choosing the one reporting the lowest variability among runs. Further accuracy was acquired by normalizing the data to the actual sample dry weight. The resulting values were expressed as metabolite relative abundance based on ion count. Statistical analyses were performed using R v3.6.1 in the RStudio environment. The Shapiro-Wilk test was used to test data for normality. Prior to the analysis of variance, Levene's and Bartlett's tests were used for assessing the homoscedasticity of variances among treatments (ANOVA assumptions). The effect of the treatments, involving two cultivars, three soils, and their interaction, was assessed by means of multifactorial ANOVA using the aov() function of the “car” package, separately for each of the annotated metabolites. To compare means between treatments found to be significantly different, Tukey's HSD test in the “agricolae” package at the 95% confidence level (P < 0.05) was used as a *post-hoc* test. The principal component analysis (PCA) was carried out using the “FactoMineR” R package. To relate the metabolites of each biological replicate with the soil features of the relative box, correlation matrices were constructed using the built‐in cor() function with the “Pearson” algorithm. Correlation matrices were visualized using the corrplot function in the “corrplot” package (Wei and Simko, 2016). Correlations and anticorrelations were considered robust when r > 0.5 or r < -0.5, respectively, and/or when statistically significant. A regression analysis of each metabolite with soil feature content was done for such metabolites as were highlighted in the previous correlations.

## Results

### Physico-Chemical Analyses of Soils

**Figure 2:**
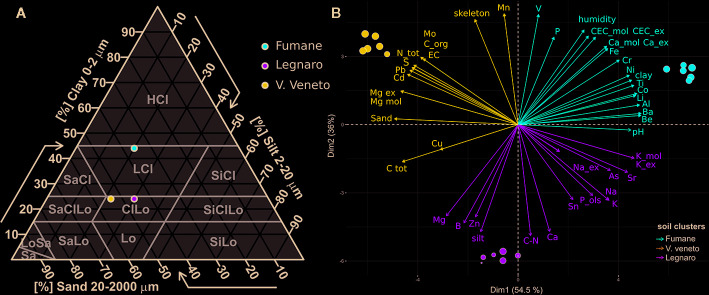
**(A)** Texture triangle according to the IUSS (International Union of Soil Science). Soils were classified based on the percentages of sand, silt, and clay. CiLO, clay loam; HCl, heavy clay. **(B)** Principal component analysis (PCA) and biplot between PC1 (Dim1) and PC2 (Dim2) showing the contribution of 48 physico-chemical parameters (including microelements, macroelements, and exchangeable cations) in explaining the variability of the graphical disposition of the three soils under study. Each colored point represents a soil replicate (n = 6). Red points refer to Fumane soil (F), blue points to Vittorio Veneto (VV), and green points indicate Legnaro soils (L).

The main soil features and their differences based on ANOVA analysis are summarized in **Table 1**, while all the other characteristics are detailed in **Supplementary Table S1**. All soils under study presented alkaline pH values ranging between 7.8 and 8.1. The skeleton, which can favor water drainage and reduce water-holding capacity, was almost absent in soil L (0.1%) but present in F (21.8%) and VV (40.0%). Other notable differences between the soils regarded texture, organic carbon content, and potassium level. The coarse fraction was much higher in soils VV (54%) and L (46%) in comparison with F (36%), which instead presented a fairly high clay level (44%). According to the IUSS, F can be classified as heavy clay soil, whereas L and VV are clay loam (**Figure 2A**). Organic carbon was three times higher in VV (3.33%) with respect to L and F. Nevertheless, soil VV presented the lowest potassium level (5.74 g/kg s.s.). Soil F was characterized by a high level of cation-exchange capacity (40.9 cmol/kg s.s.), followed by VV and L, whose levels were lower (32.7 and 21.9 cmol/kg s.s., respectively). The Principal Component Analysis (PCA) based on soil physico-chemical characteristics clearly clustered replicates according to their origin (**Figure 2B**). PC1 explained 54.5% of the total variance and divided VV from F well. PC2 explained 36% of the variance, clearly separating soils F and VV from L. The biplot graph (**Figure 2B**) also indicates the contribution of each soil characteristic to the soil grouping disposition. As concerns the PC1, amongst the most discriminant components of agronomical relevance were sand, exchangeable magnesium and potassium, clay, total nitrogen, organic carbon, and electric conductivity. Conversely, manganese, silt, calcium, skeleton, boron, cation exchange capacity, humidity, phosphorus, exchangeable calcium, and iron were the most discriminant elements for PC2. **Supplementary Figure S1** reports the PCA obtained using both physio-chemical characteristics of soils in the caissons and those in the areas of origin (“on site”).

**Table 1:** Main soil physico-chemical characteristics of VV, L, and F soils.

		Legnaro (L)	Fumane (F)	V. Veneto (VV)
*Texture*				
	Sand (%)	46.000	36.000	54.000
	Silt (%)	30.000	20.000	22.000
	Clay (%)	24.000	44.000	24.000
*Skeleton*		0.082	21.775	39.979
*Macroelements*				
	Ca (g/kg s.s.)	81.896 ± 1.392 a	52.018 ± 1.443 b	34.263 ± 2.230 c
	Fe (g/kg s.s.)	27.346 ± 0.196 c	36.904 ± 0.426 a	30.631 ± 0.227 b
	K (g/kg s.s.)	15.254 ± 0.305 a	14.178 ± 0.123 b	5.740 ± 0.056 c
	Mg (g/kg s.s.)	37.329 ± 0.404 a	8.391 ± 0.090 c	22.356 ± 0.875 b
	Mn (g/kg s.s.)	0.662 ± 0.004 c	1.243 ± 0.032 b	1.424 ± 0.015 a
	Na (g/kg s.s.)	0.897 ± 0.031 a	0.837 ± 0.013 a	0.371 ± 0.007 b
	P (g/kg s.s.)	0.898 ± 0.008 b	1.005 ± 0.020 a	0.978 ± 0.010 a
	S (g/kg s.s.)	0.221 ± 0.005 b	0.161 ± 0.002 c	0.571 ± 0.006 a
*Microelements*				
	B (mg/kg s.s.)	25.112 ± 0.141 a	20.223 ± 0.231 c	21.944 ± 0.139 b
	Ba (mg/kg s.s.)	286.234 ± 6.871 b	525.950 ± 5.616 a	178.995 ± 1.460 c
	Be (mg/kg s.s.)	1.717 ± 0.015 b	2.213 ± 0.022 a	1.475 ± 0.014 c
	Mo (mg/kg s.s.)	nd	0.021 ± 0.021 b	1.646 ± 0.068 a
	Zn (mg/kg s.s.)	103.694 ± 1.513 a	92.660 ± 0.934 b	95.728 ± 0.849 b
*Others*				
	CEC (mg/kg s.s.)	4258.192 ± 71.587 c	8224.210 ± 74.034 a	6126.449 ± 112.854 b
	pH	7.903 ± 0.015 b	8.083 ± 0.026 a	7.767 ± 0.038 c
	OC (% s.s.)	1.113 ± 0.029 b	0.892 ± 0.021 c	3.333 ± 0.086 a
	P-Ols (mg/kg s.s.)	34.193 ± 0.751 a	31.599 ± 2.225 a	20.291 ± 1.373 b

Values are reported as mean ± standard error (n = 6). Letters indicate significant difference according to Tukey's post-hoc test (P < 0.05).

### Berry Phenology and Physiology Indicate Different Plant Response to Soil Effect

Throughout the vegetative growth, statistical analysis did not reveal any significant effect of the soil factor on the canopy traits considered (LAIe and shoot length; **Table 2**). No interaction effect (soil × cultivar) was detected either. As concerns berry phenology and physiology, grapes collected at the three sampling dates corresponding to complete véraison (36 E-L), mid-ripening (37 E-L), and harvest (38 E-L), revealed no significant differences among soils in pH and titratable acidity (**Table 2**). Conversely, the two-way ANOVA with soil and variety as factors revealed that sugar content (°Brix) was significantly affected by different soils at both complete véraison and mid-ripening stages, with plants grown in F showing higher values compared to those in VV and L. Despite such differences emerging, the phenological development (Coombe, 1995) remained comparable among soils and between cultivars. Such differences faded throughout ripening, becoming far from significant at harvest. As expected, the “variety” factor was statistically significant at all-time points considered, with Corvina always showing higher Brix values than Glera (data not shown). Nonetheless, the phenological development between varieties was comparable. Similar to what was observed for the TSS content, the maturation index (MI) was also statistically significant at complete véraison and mid-ripening as concerns the soil factor, with plants grown in Legnaro soil having the lowest values. Such differences were not observed at harvest. In addition, berry weight at harvest was not affected by the soil factor. Also, for the berry physiological parameters, ANOVA did not reveal any statistical significance for the soil × cultivar interaction.

**Table 2:** Canopy and berry physiological parameters.

	Date	Legnaro (L)	Fumane (F)	V. Veneto (VV)
*Shoot length (m)*				
	27/04/2018	45.50 ± 2.11	47.54 ± 1.93	43.26 ± 2.35
	02/05/2018	72.20 ± 1.90	69.83 ± 2.17	67.22 ± 2.73
	07/05/2018	101.41 ± 3.58	97.88 ± 3.57	96.25 ± 3.69
	14/05/2018	133.12 ± 4.54	126.19 ± 4.41	123.73 ± 4.89
	24/05/2018	176.24 ± 5.58	161.33 ± 6.32	162.57 ± 7.28
*LAIe*				
	24/05/2018	1.10 ± 0.07	1.11 ± 0.05	1.09 ± 0.06
	20/06/2018	1.25 ± 0.08	1.26 ± 0.05	1.32 ± 0.07
	28/06/2018	1.28 ± 0.08	1.29 ± 0.05	1.38 ± 0.08
	03/07/2018	1.53 ± 0.11	1.32 ± 0.07	1.52 ± 0.08
	13/07/2018	1.08 ± 0.07	1.11 ± 0.04	1.08 ± 0.06
	20/07/2018	1.09 ± 0.08	1.12 ± 0.04	1.13 ± 0.05
*Sugar (°Brix)*				
(T1)	16/08/2018	13.74 ± 0.35 b	15.33 ± 0.37 a	14.20 ± 0.35 b
(T2)	29/08/2018	17.18 ± 0.34 b	18.06 ± 0.31 a	17.53 ± 0.27 ab
(T3)	11/09/2018	17.95 ± 0.48	18.76 ± 0.43	18.35 ± 0.45
*pH*				
	16/08/2018	3.52 ± 0.01	3.52 ± 0.03	3.49 ± 0.02
	29/08/2018	3.38 ± 0.04	3.41 ± 0.04	3.34 ± 0.04
	11/09/2018	3.46 ± 0.05	3.48 ± 0.04	3.44 ± 0.03
*Titratable acidity (g/L)*				
	16/08/2018	8.15 ± 0.18	8.49 ± 1.52	6.95 ± 0.61
	29/08/2018	5.87 ± 0.42	5.31 ± 0.45	5.30 ± 0.47
	11/09/2018	4.84 ± 0.52	4.62 ± 0.33	4.75 ± 0.31
*Maturation index (MI)*				
	16/08/2018	1.68 ± 0.04 b	2.02 ± 0.13 a	2.09 ± 0.10 a
	29/08/2018	3.03 ± 0.14 b	3.49 ± 0.11 a	3.42 ± 0.14 a
	11/09/2018	4.02 ± 0.27	4.16 ± 0.15	3.97 ± 0.14
*Berry weight (g)*				
	11/09/2018	1.81 ± 0.10	1.74 ± 0.09	1.64 ± 0.07

Values are reported as mean ± standard error (n = 6). Letters indicate significant difference according to Tukey's post-hoc test (P < 0.05).

### Soil Affects the Accumulation of Stilbenes, Flavonols, and Hydroxycinnamic Acids

**Figure 3:**
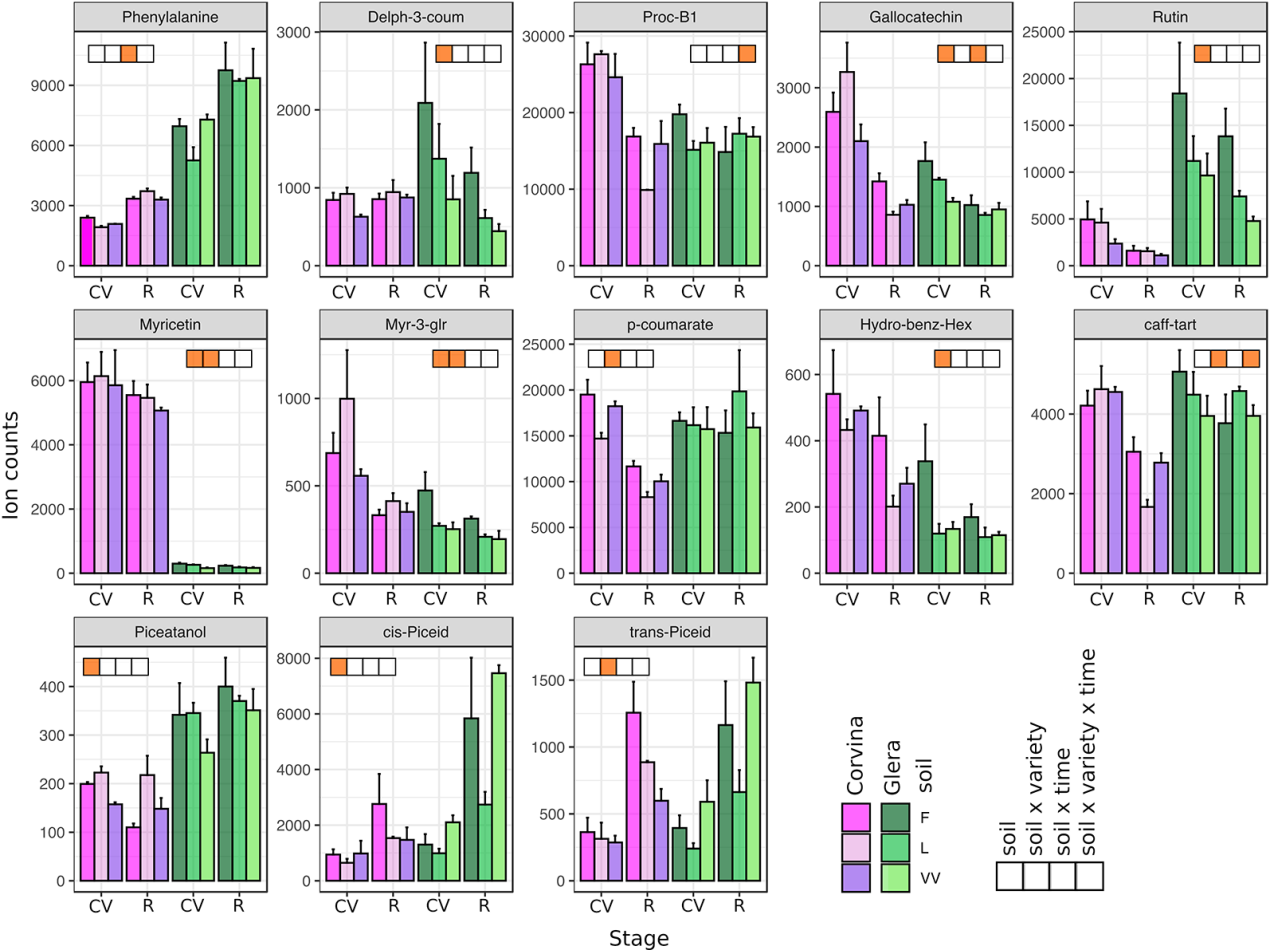
Secondary metabolites detected by LC-MS in berry skin tissues in response to different soils. Included are metabolites found to be significantly affected by the soil factor and its interactions with other factors (variety and phenological stage) based on multifactorial ANOVA. Levels represent relative abundance based on ion counts. Purple bars represent the Corvina variety and green bars represent the Glera variety. Different color intensities indicate different soils, whereas orange squares indicate the statistical significance related to soil factor and/or its interactions with the other factors. CV and R stand for complete véraison and ripening, respectively. Error bars indicate standard error (n = 3).

**Figure 4:**
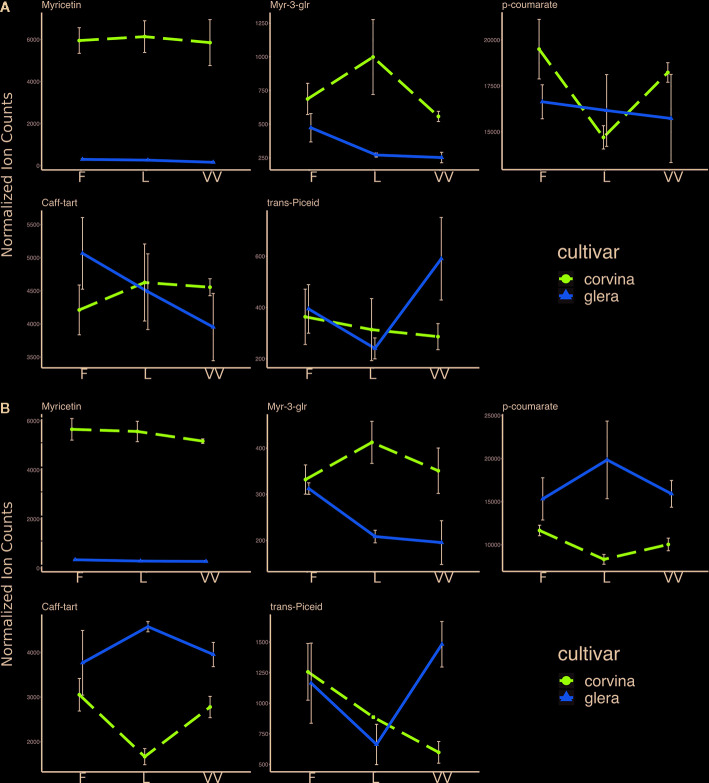
Significant metabolites modulated by Genotype × Environment interactions in Glera and Corvina. Levels represent relative abundance based on ion counts. Purple lines represent Corvina variety, and yellow lines represent Glera variety. **(A)** G×E interactions at complete-véraison (CV) stage; **(B)** G×E interaction at ripening (R) stage. Error bars indicate standard error (n = 3).

By using a targeted approach, 52 metabolites were analyzed, normalized to the internal standards and dry weight, and subjected to statistical analyses. The m/z and retention time values of detected metabolites are reported in **Supplementary Table S2**. Most of the detected metabolites belonged to the secondary metabolism, including the amino acids tryptophan and phenylalanine, one flavanonol, one flavanone, some anthocyanins (mainly limited to Corvina variety), flavan-3-ols (proanthocyanins), flavonols, hydroxycinnamic acids, and stilbenes. A comprehensive ANOVA on both cultivars and both phenological stages allowed the identification of 13 metabolites significantly affected by the soil factor and/or by its interaction with the other factors considered (**Figure 3**). The soil factor significantly affected three out of 10 detected flavonols (rutin, myricetin, and myr-3-glu) and two out of six stilbenes (piceatannol, *cis*-piceid). Also, *trans*-piceid appeared to be modulated by the soil factor, even though this was not statistically confirmed (P = 0.067). Other differences regarded the amount of anthocyanin delph-3-coum, flavanol gallocatechin, and hydroxybenzoate hex. The soil factor also seemed to influence the skin tartaric acid content slightly (P = 0.059). Generally, soil F was often associated with the highest level of metabolites in berry skin, whereas VV always showed the lowest ranking. This was not true in stilbenes, where the highest accumulation of piceatannol was observed in soil L, whereas *cis*-piceid content was higher in berry skins from plants grown in soil VV (**Supplementary Table S3**). Metabolite accumulation was also affected by the interaction between multiple experimental factors. The specific pattern of accumulation of several metabolites depended not only on the soil where plants were grown but also on its interaction with the specific variety and phenological stage considered. Soil × cultivar interaction (**Figure 4**) mainly concerned the content of flavonols (myricetin and myr-3-glr), some acids (p-coumarate and caff-tart), and the stilbene *trans*-piceid. Interestingly, p-coumarate and caff-tart contents were not significantly affected by the singular soil factor but only by soil × cultivar interaction. Phenylalanine and gallocatechin contents were found to be strongly affected by the interaction between soil and phenological stage (**Figure 3**). The analysis of variance allowed us to focus also on the effect resulting from the interaction of all of the three factors considered: soil, cultivar, and phenological stage (i.e., soil x cultivar x phenological stage). In this case, there was a statistical significance for metabolite content related to proc-B1 and caff-tart compounds (**Figure 3**).

Considering that most of the anthocyanins were not included in the comprehensive ANOVA since they were not detected in Glera variety, we conducted a new analysis separating the singular varieties and the two phenological stages (complete véraison and ripening). Moreover, this approach allowed us to investigate in more detail the meaning of all interactions identified in the general analysis. **Table 3** reports all statistically significant metabolites with related p-values in the different analyses performed. ANOVA revealed that the R stage is generally more affected by the soil factor than the CV stage and that Corvina secondary metabolites seem to be more plastic than those of Glera (**Supplementary Tables S4** and **S5**). At complete véraison (CV) in both varieties, only three metabolites were significantly affected by soil: phenylalanine, gallocatechin, and myricetin in Glera and phenylalanine, p-coumarate, and piceatannol in Corvina. Thus, the white variety responded to different soils by modulating the accumulation of flavanol and flavonol compounds, together with phenylalanine, which is the basic unit of all phenylpropanoids, whereas Corvina preferentially modulated the biosynthetic pathway related to stilbenes, of which both phenylalanine and p-coumarate represent precursors (**Supplementary Table S4**). At the ripening stage, the Glera variety continued to modulate flavonol compounds, with rutin and Myr-3-glr being significant in the ANOVA, whereas many phenylpropanoid groups were affected in Corvina, including anthocyanins (Delph-3-coum), flavanols (Proc-B1 and gallocatechin), hydroxycinnamic acids (p-coumarate and Caff-tart), stilbenes (piceatannol and piceid), and finally tartarate (**Supplementary Table S5**).

**Table 3:** Metabolites found to be significantly affected by soil factor when separating phenological stage and variety.

	Glera CV	Corvina CV	Glera R	Corvina R
Phenylalanine	0.042	0.005	*ns*	*ns*
Tryptophan	*ns*	*ns*	*ns*	0.011
Peo-3-glu	*ns*	*ns*	*ns*	0.048
Proc B1	*ns*	*ns*	*ns*	0.048
Gallocatechin	0.050	*ns*	ns	0.016
Rutin	*ns*	*ns*	0.012	*ns*
Myricetin	0.013	*ns*	*ns*	*ns*
Myr-3-glr	*ns*	*ns*	0.040	*ns*
p-coumarate	*ns*	0.031	*ns*	0.028
Caff-tart			*ns*	0.025
Piceatanol	ns	0.002	*ns*	0.042
trans-Piceid	*ns*	*ns*	*ns*	0.021
Tartarate	*ns*	*ns*	*ns*	0.036

For each metabolite, p-values obtained by ANOVA analyses performed on different datasets are reported. ns, non-significant (P > 0.05); CV, complete véraison; R, ripening.

### Correlation Analyses Indicate Key Soil Parameters Shaping Berry Biochemical Plasticity

**Figure 5:**
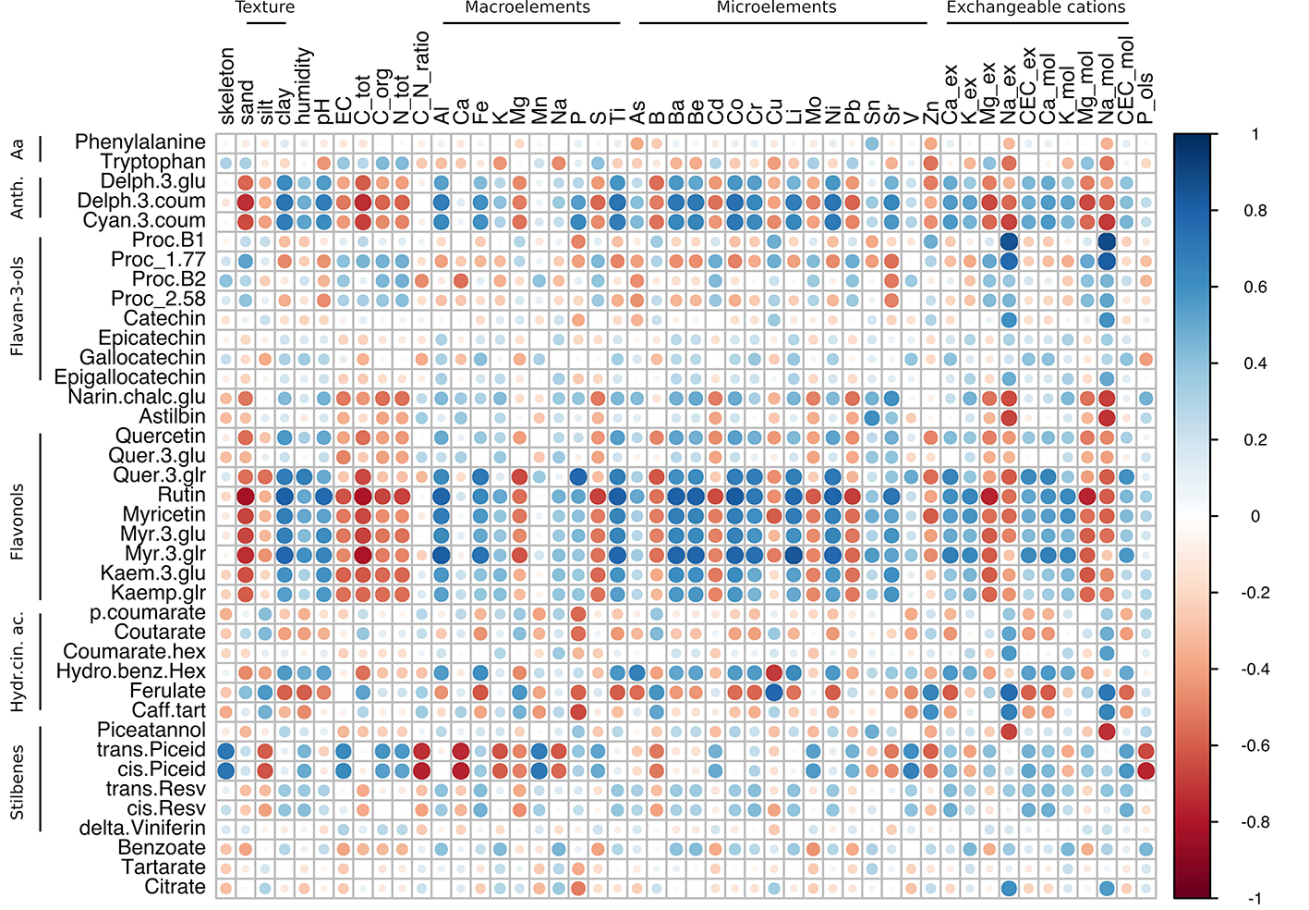
Correlation analysis of grape skin secondary metabolites of cv. Glera at harvest against the physico-chemical characteristics of the soil. The analysis was generated using Pearson's correlation.

**Figure 6:**
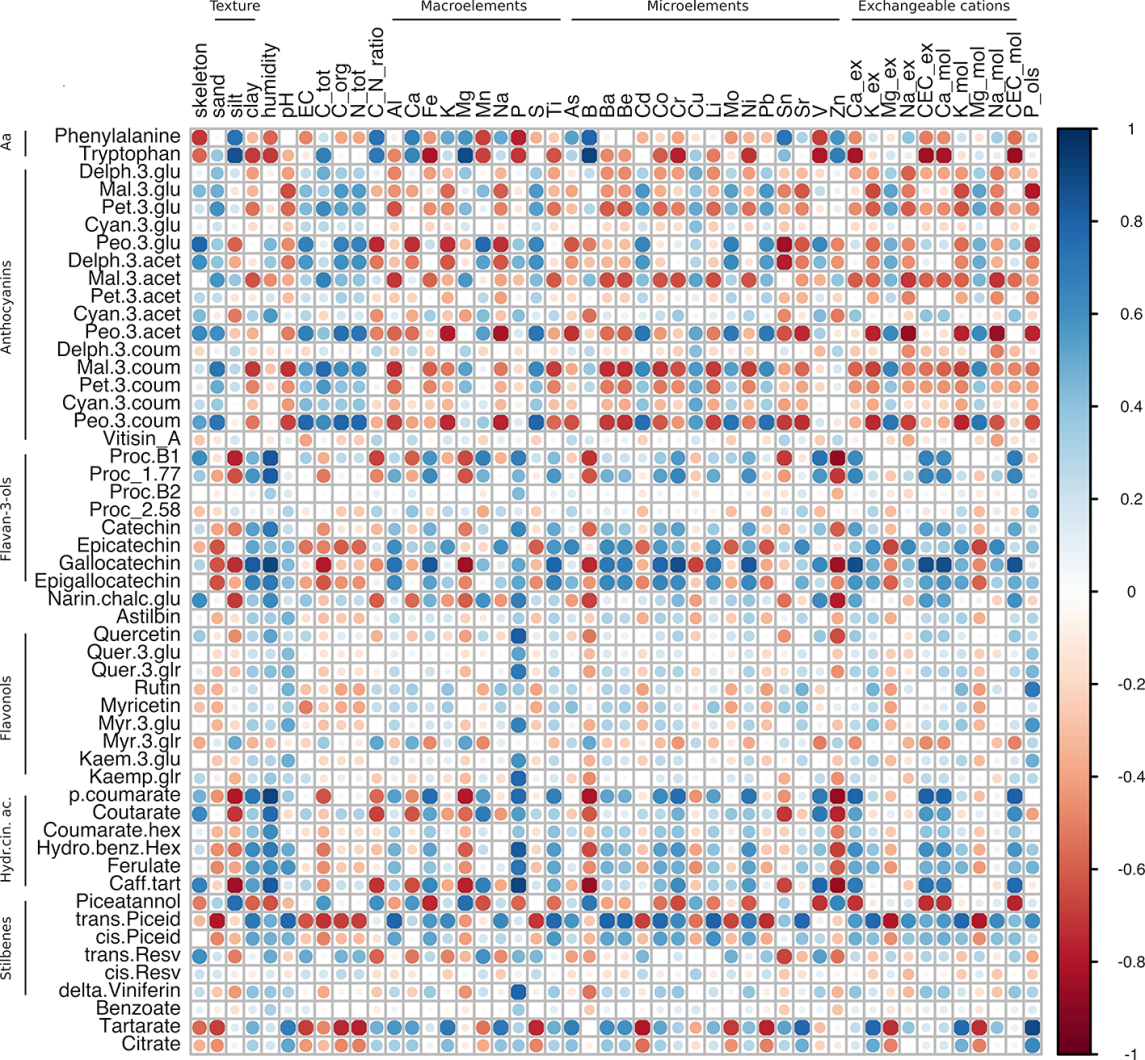
Correlation analysis of grape skin secondary metabolites of cv. Corvina at harvest against the physico-chemical characteristics of the soil. The analysis was generated using Pearson's correlation.

**Figure 7:**
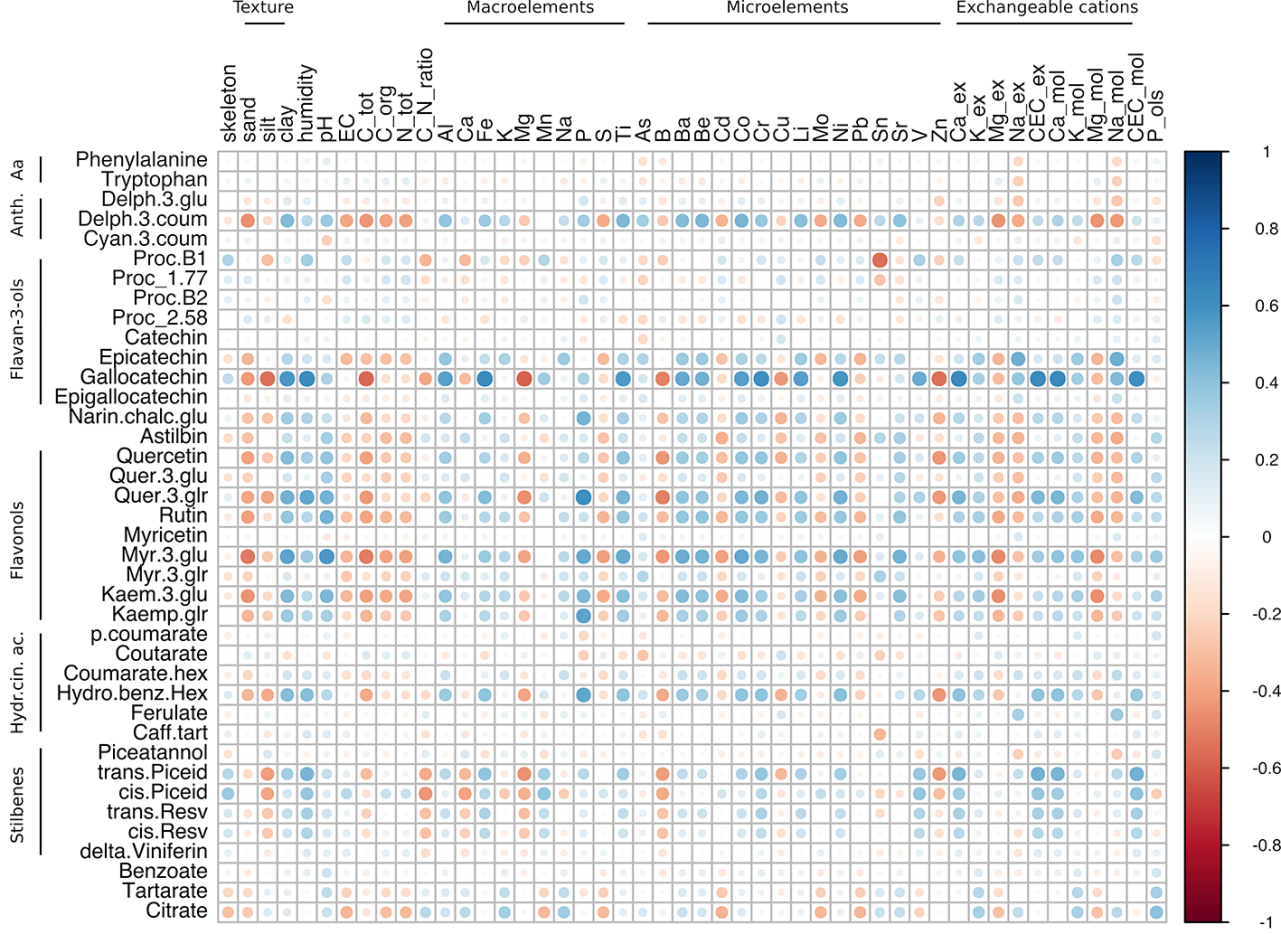
Correlation analysis of grape skin secondary metabolites of both cv. Corvina and cv. Glera at harvest against the physico-chemical characteristics of the soil. The analysis was limited to 39 shared metabolites out of 51. Most of the anthocyanins were excluded from the analysis since they accumulated exclusively in Corvina. The analysis was generated using Pearson's correlation.

To elucidate any possible relationship between the physico-chemical features of soil and the skin metabolites accumulated at harvest in the two varieties, three correlation matrices were constructed starting from Glera, Corvina, and the merged dataset. This approach allowed us to investigate and compare within-cultivar correlations (**Figures 5** and **6**) but also to investigate a higher level of correlation regardless of the variety considered (**Figure 7**). The within-cultivar pairwise correlations (|r| > 0.5) detected in Corvina were higher in number compared to those detected in Glera, whereas only a few of them were found in the merged dataset (Corvina and Glera). In all cases, the number of negative and positive correlations was comparable and mainly involved entire classes of metabolites.

The within-variety correlation matrix in Glera highlighted correlations between anthocyanin, flavonol, and stilbene compounds and specific soil characteristics (**Figure 5**; **Supplementary Table S6**, Sheet 1). The anthocyanin delph-3-glu, delph-3-coum, and cyan-3-coum and the flavonols quercetin, rutin, myricetin (all forms), and kaempferol (all forms) were positively correlated (r > 0.5) with the clay content, humidity, pH, Al, Fe, P, Ti, Ba, Be, Co, Cr, Li, Ni, Sr, and ex-Ca. Conversely, these metabolites were negatively correlated (r < -0.5 in most cases) with sand, EC, total carbon, organic carbon, total N, Mg, S, B, ex-Mg, and ex-Na. As concerns the stilbene group, *trans*- and *cis*-piceid were correlated with skeleton, EC, organic C, total N, Mn, Mo, V, and CEC, whereas anticorrelations were observed with silt, C/N ratio, Ca, K, Mg, Na, B, Sr, Zn, and P-Olsen. Other interesting correlations regarded the amino acid tryptophan with Zn and Na-ex (-0.53 and -0.55, respectively) and the procyanidin B1 and catechin, which positively correlated with ex-Na (0.86 and 0.60 respectively). Overall, exchangeable sodium (Na-ex) was the soil parameter showing the highest number of correlations (17 out of 39), with negative values with anthocyanins, flavanones, flavanonols, flavonols, and stilbene groups and positive values with hydroxycinnamic acids and flavanols (catechins).

Looking at the Corvina within-variety correlation matrix, all secondary metabolite groups were found to be highly correlated with many soil components, except for flavonols (the yellow pigments), which showed positive correlations only with P content (**Figure 6**; **Supplementary Table S6**, Sheet 2). We observed similar and/or opposite correlation patterns between different secondary metabolite groups. In general, flavanols and hydroxycinnamic acids showed similar behavior, being positively and negatively correlated with the same soil parameters. Conversely, anthocyanins often showed the opposite behavior. To summarize, soil parameters were divided into i) parameters showing opposite behavior (both positive and negative correlations) between flavanols/hydroxycinnamic acids and anthocyanins; ii) parameters showing strong correlations with flavanols and hydroxycinnamic acids; iii) parameters showing strong correlations with anthocyanins. Those soil parameters belonging to group (i) were clay, several macro- (Fe, Al, Ti) and microelements (Ba, Be, Co, Cr, Li, Ni, Sr), and most of the exchangeable bases, which showed positive correlations with flavanols and hydroxycinnamic acids and negative correlation with anthocyanins. Conversely, sand, total C, Mg, Cu, Pb, Mg-ex, and Mg-mol, showed a positive correlation with anthocyanins and negative correlations with flavanols and HA. Among those parameters showing positive correlations (r > 0.5) with flavanols and hydroxycinnamic acids but not with anthocyanins (ii) were skeleton, humidity, P, and V, whereas silt, C/N ratio, Ca, B, Sn, and Zn showed negative correlations. Finally, looking at those parameters showing good correlation with anthocyanins (|r| > 0.5) but not with flavanols (|r| < 0,5) (iii), EC, C-org, N-tot, S, Cd, and Mo, were all positively correlated and K, Na, Sn, and P-Olsen, were negatively correlated.

In comparison with the other groups analyzed, stilbenes did not follow any specific trend. Among them, those metabolites showing the highest correlation coefficients were piceatannol and piceid (both *trans* and *cis* forms). In most cases, these complex stilbenes showed an opposite trend between them. Piceatannol highly correlated with skeleton, clay, humidity, macroelements including Fe, Mn, P, and Ti, the microelements Co, Cr, Li, Ni, and V, and the cation exchange properties related to Ca-ex, CEC-ex, Ca-mol, and CEC-mol. Negative correlations were limited to silt, total C, C/N ratio, Ca, Mg, B, Cu, and Zn. Conversely, piceid positively correlated with clay, humidity, pH, Al, Fe, K, Na, Ti, As, Ba, Be, Co, Cr, Li, Ni, Sr, K-ex, Na-ex, CEC-ex, Ca-mol, K-mol, Na-mol, and P_ols and negatively correlated with sand, EC, total C, organic C, total N, S, Cd, Cu, Mo, Pb, Mg-ex, and Mg-mol. It is worth mentioning the strong anticorrelations between *trans*-resveratrol and Sn (r = -0.67). Delta-viniferin had a strong positive correlation with P (r = 0.78). Phosphorous (P) was the soil element with the highest number of strong correlations (19 out of 52). In particular, it never correlated with anthocyanins but did correlate positively with the flavanones, some flavanols (procyanidin B1, catechin, and gallocatechin), many flavonols and hydroxycinnamic acids, and the stilbene delta-viniferin. Negative correlations were found between P and piceatannol and the amino acids phenylalanine and tryptophan.

### Combined Dataset

When correlating soil components with the Glera and Corvina combined dataset (**Figure 7**; **Supplementary Table S6**, Sheet 3), the number of significant correlations observed was considerably lower than those resulting from the two separated datasets, with the flavonol group being the one accounting for the highest number of significant correlations. In detail, quer-3-glr and myr-3-glu were characterized by positive correlations with clay, P, and Ni. Moreover, myr-3-glu was strongly positively correlated with pH, Ti, Ba, Be, and Co, but negatively correlated with sand, total C, Mg-ex, and Mg-mol, whereas quer-3-glu showed specific positive correlations with humidity and Cr but negative correlations with B. The flavanol gallocatechin behaved similarly to flavonol metabolites, positively correlating with clay, humidity, Ti, Ba, Be, Co, Cr, and Ni, and negatively with total C and B. Moreover, it was found to be enriched with many other strong anticorrelations such as with silt, Mg, and Zn and positive correlations with Al, Fe, Li, V, Ca-ex, CEC-ex, Ca-mol, and CEC-mol. Other significant correlations identified regarded procyanidin B1 with Sn (r = -0.56), epicatechin with Na-ex and Na-mol (r = 0.48 and 0.49 respectively), and *trans*-piceid with CEC-ex and CEC-mol (r = 0.47 and 0.48 respectively). Indeed, among the detected stilbenes, the two piceid forms (*trans* and *cis*) were those found to harbor the highest correlation values with soil features. They also tended to negatively correlate with silt, Mg, B, and Zn, although this was not statistically confirmed. It is worth noticing that, among the soil components, P was the element showing the highest number of strong correlations (only positive ones). As well as with the already mentioned flavonols (quer-3-glu, myr-3-glu), P also correlated with kaemp-glr, the flavanone narin-chalc-glu, and the hydroxycinnamic acid hydroxy-benz-hex.

### Relations Between Results Obtained From ANOVA and Correlation Analyses

An important observation is that all those metabolites showing the highest number of significant correlations with soil parameters (**Supplementary Table S6**) are also among those found to be significantly modulated by the soil factor (**Supplementary Table S5**). This applies to both varieties, in which the ANOVA was also carried out separately for the two datasets limitedly to harvest point (38 E-L). In detail, among Corvina metabolites are the amino acids phenylalanine and tryptophan, the anthocyanins peo-3-glu, peo-3-acet, mal-3-coum, and peo-3-coum, the flavan-3-ols proc-B1 and gallocatechin, the hydroxycinnamic acids p-coumarate, coutarate, and caff-tart, the complex stilbenes piceatannol and trans-piceid, and, finally, tartaric acid. Among Glera metabolites are the anthocyanin delph-3-coum and the flavonols quer-3-glr, rutin, and myr-3-glr (P < 0.1; **Supplementary Table S5**).

**Figure 8:**
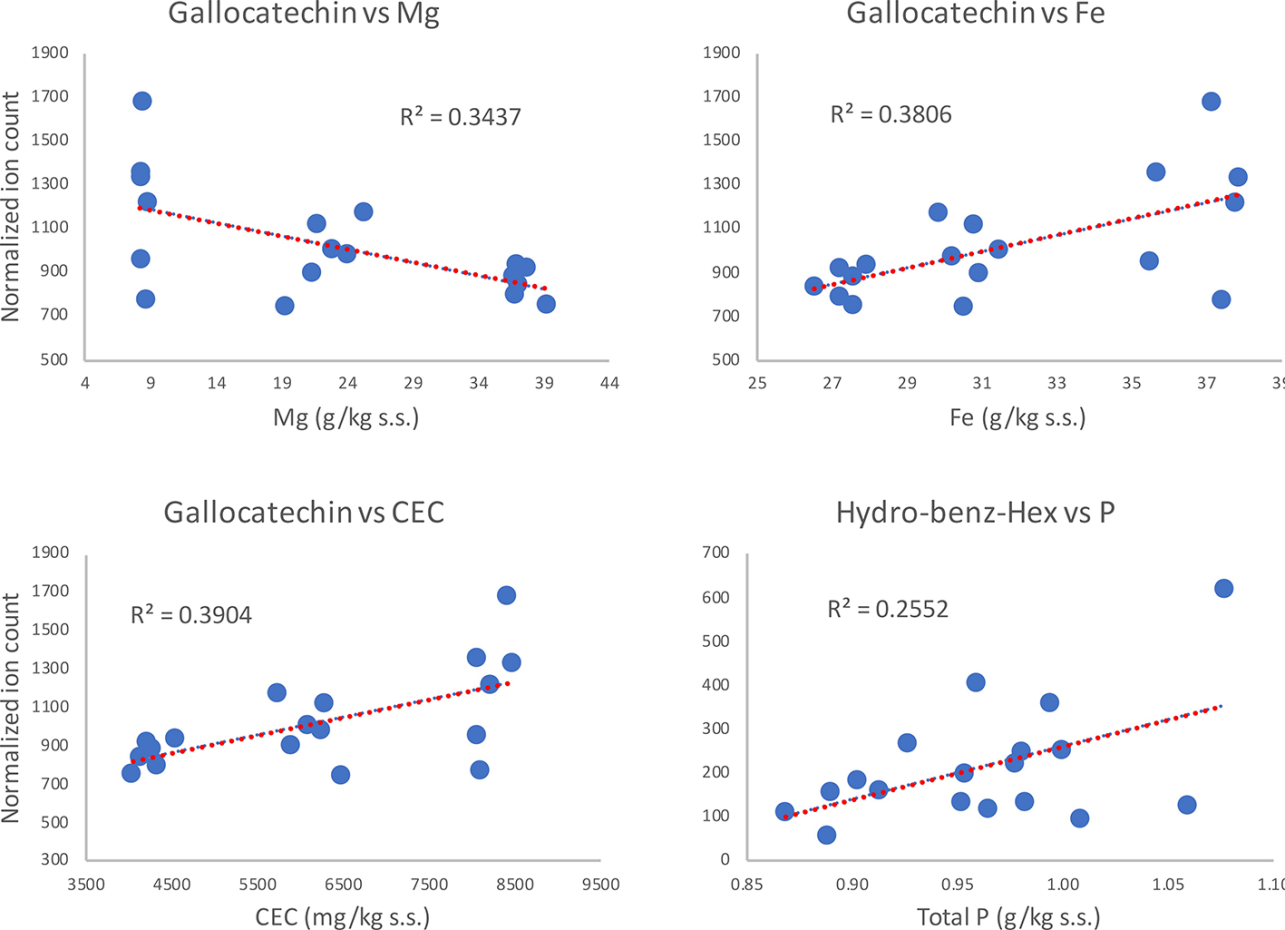
Regression analysis of grape metabolites at harvest (ripening; R) significantly correlating (P > 0.05) with soil features, independently of the cultivar.

Interestingly, such observations were not valid in the combined dataset (Corvina + Glera at harvest) where, among the metabolites differently modulated by soil factor (i.e., the flavanol gallocatechin, the flavonol rutin, the acid hydroxy-benz-hex, and the stilbene *cis*-piceid; **Supplementary Table S5**), only gallocatechin showed positive correlations (P < 0.05), with clay, humidity, Al, Fe, Ti, Ba, Be, Co, Cr, Li, Ni, V, Ca_ex, and CEC, and negative correlations with silt, total C, Mg, and Zn (**Supplementary Table S6**, Sheet 3). Even the positive correlation between hydro-benz-hex and soil P was supported by statistical significance (p-value = 0.033). Instead, the fact that rutin and *cis*-piceid contents were not characterized by strong correlations suggests that such differences in metabolite amounts might not be attributed to differences in soil mineral composition. On the other hand, the observations regarding gallocatechin and hydroxy-benz-hex might lead one to think about a direct involvement of soil mineral composition in skin metabolite accumulation (**Figure 8**).

## Discussion

Among the multiplicity of factors affecting grape quality and, consequently, wine value and appreciation, the secondary metabolites of grape play an extremely important role in shaping the sensorial profile and experience of the derived product. In addition to their contribution in determining the final organoleptic and olfactory properties of wine, these substances are extremely important also from a medical point of view, with an increasing number of studies revealing that compounds such as stilbenes and flavonoids provide many potential benefits for human health (Guerrero et al., 2009; Carrieri et al., 2013). The production of secondary metabolites, mainly phenylpropanoids, which typically accumulate in berry exocarp, is known to be sensitive to external environmental conditions (Ali et al., 2010). This observation led to an increasing interest in studying berry composition and its modulation as a response to different terroir factors (Swiegers et al., 2005; Lund and Bohlmann, 2006; Kuhn et al., 2013) including the choice of variety, the climate, and the soil (Bautista-Ortin et al., 2012). The latter factor, in particular, has long been known for its determining role in shaping the grape biochemical composition (Coelho et al., 2006; Vilanova et al., 2007; Ubalde et al., 2010), although only a few studies have been conducted so far trying to isolate its specific and singular effect (Seguin, 1986; Van Leeuwen et al., 2004).

The present study, which can be considered the first of its kind, was aimed at shedding light onto this particular issue, trying to dissect the effect of the soil factor on berry skin biochemical plasticity in two grapevine varieties that are of great importance at both the national and international level: Glera and Corvina. With this aim, in order to isolate the soil effect and to keep all other variables as stable as possible, both varieties were grown in concrete boxes located in a limited area at the experimental farm of the University of Padova that were filled with three different soils collected from three important viticultural areas within the Veneto region: the DOCG Prosecco area (VV), the DOCG Corvina area (F), and the experimental farm of the University of Padova (L), which is located within the border of the Prosecco DOC areal. Due consideration must be given to the fact that the mobilization and transport of soils led to the loss of their original structure and stratification. Nonetheless, the aim of the present study was related to the effect of different substrates on the plasticity of secondary metabolites regardless of whether they faithfully reflected the horizon of the soils in the localities of origin. Both the Glera and Corvina varieties were grafted onto the same rootstock, Kober 5BB, chosen because it is known to ensure root mineral absorption in the presence of alkaline soils and active limestone (http://vivairauscedo.com/en/portinnesti) and it shows comparable performance in many different soils and graft combinations, avoiding the introduction of biases into the experimental design due to differences in the performances of different soil/rootstock or scion/rootstock combinations. All soils considered in our trial were alkaline (pH > 7.5), allowing their mineral supply to be compared just based on the quantification of elements and not on their biological availability, which mainly depends on pH. Although the three soils can be considered as very different, mainly due to skeleton content and textural composition (which influence the water holding capacity of the soil as well as mineral availability), they have been selected in this study for their long-lasting shared history of grapevine production. Among the elements that better separated our soils in the PCA, K, organic C, Fe, and Cu were reported to exert a strong effect on the mineral composition of grapevine leaves (Likar et al., 2015), which in turn, as shown by Peuke (2009), can influence grape mineral allocation as well as metabolite accumulation throughout ripening.

On the one hand, the study aimed to identify those metabolites that appear to be modulated in response to different soils regardless of the cultivar involved or in a cultivar-dependent manner. Furthermore, we tried to go deeper into the relationships between skin metabolite accumulation and soil physico-chemical properties. As complementary information, we also collected measures related to the phenological and physiological behavior of plants grown in different soils.

When compared with the 2017 season (Perin et al., 2019), berry physiology in 2018 showed lower plasticity in response to the soil factor. A possible explanation of such differences could be the fact that the effect size of soil factor on berry development might not be so strong as to determine differences in every year, and this might be affected by interaction with other terroir variables including the plant age and the meteorological features of the season. Though some differences emerged at complete véraison, Brix values were generally comparable (i.e., no differences in phenological development), allowing us to directly compare samples collected on the same date (**Table 2**).

The ANOVA on secondary metabolites accumulated by both varieties in the three soils indicated several plastic compounds specifically modulated by the soil factor. Although pedoclimatic conditions were already reported to strongly influence the ripening dynamics in terms of phenolic biosynthesis (Dal Santo et al., 2016), in the present study, this effect could be directly related to the singular soil factor, excluding any other environmental or agronomical variable. Among phenylpropanoids, flavonols and stilbenes were the two most affected groups. Rutin and myricetin were identified as the most plastic compounds, as supported by statistics when considering both varieties together but also at the intra-varietal level, limited to Glera. Thus, beyond light (Cortell and Kennedy, 2006; Reshef et al., 2017), temperature (Del-Castillo-Alonso et al., 2016), UV-B radiation, and biotic stresses (Kuhn et al., 2013), their accumulation has now been linked to the soil factor. The lack of any water stress and the observations that soil L was characterized by a high water retention capacity and was also the one with the lowest flavonol content exclude the possibility of experimental bias related to possible collinearity between the soil factor and water deficit (Kuhn et al., 2013). The results related to stilbenes are consistent with previous studies showing the high plasticity of these phytoalexins (Anesi et al., 2015). ANOVA showed that piceid and piceatannol were significantly modulated by the soil factor in our experimental trial, with Corvina showing higher plasticity in piceatannol content and Glera in piceid content when the two varieties were considered separately (**Supplementary Table S3**). The capability of the soil to influence the stilbene biosynthesis was already reported by Dal Santo et al. (2016), who described a high variability in *trans*-resveratrol accumulation in berries of Corvina grown in different locations. Moreover, the soil factor has been proposed to be as important as the climate for the stilbene amount in berries (de Andrés-de Prado et al., 2007), and this hypothesis found confirmation in this study.

Metabolites from the two groups herein described (flavonols and stilbenes) were not only reported to be affected by the soil factor but also by the interaction between soil and genotype (**Figure 4**, **Supplementary Table S3**). In other words, in a given variety, the amount of a certain metabolite had a specific trend among different soils, which was not the same in the other cultivar. This observation supports the hypothesis of genotype-dependent metabolic plasticity in response to soil factor, which, from an enological point of view, represents the biochemical basis of terroir. A particular mention concerning the role of the genotype in the secondary metabolite profile regards anthocyanins, which are typically accumulated in the red varieties (Corvina). Based on our specific study, these compounds showed moderate responsiveness to soil factor, although the peonidin forms (peo-3-glu, peo-3-acet, and peo-3-coum) and myr-3-coum showed p-values close to statistical significance. If this observation is in agreement with many other studies reporting that anthocyanin accumulation is more related to climatic conditions (e.g., Ubalde et al., 2010), caution is needed when considering the role of soil in the accumulation of this class of compounds due to the limited number of soils and plants considered in our experimental plan.

Contrary to Glera, many Corvina metabolites were also affected by the interaction between soil and phenological stage (complete veraison – ripening/harvest), suggesting that different varieties accumulate metabolites according to their kinetics. As a consequence, the influence of the external environment might differ depending on the timing throughout berry maturation. Dal Santo et al. (2013) already proposed the existence of high plasticity of Corvina berries at harvest and a high potential impact of other terroir factors than climate on berry transcriptome at ripening. Instead, Glera metabolites seemed more affected by the singular effect of the soil factor than by its interaction with the stage, somehow narrowing the array of possible phenotypes and potentially reducing its plasticity. A high responsiveness to pedoclimatic conditions was already reported for another white variety, *V. vinifera* cv. Garganega, in terms of phenolic compound accumulation during the overall ripening period in different environments (Dal Santo et al., 2016).

With the aim of isolating those soil characteristics that better explain the biochemical plasticity of berry skin in the two varieties under study, we tried to investigate the correlations existing between soil features and berry metabolites at harvest. The number of good correlations was higher when the two cultivars were considered separately. This observation could depend on the different statistical power of the two analyses but could also indicate that the biochemical plasticity of berries in response to soil composition deeply depends on the genotype. This concept is also supported by the number of significant ‘soil × cultivar' interactions revealed by the analysis of variance. Most of the correlations identified regarded red pigments (anthocyanins) in Corvina, yellow pigments (flavonols) in Glera, and stilbenes in both varieties. One of the best correlating soil features was the clay content, which seemed to have a strong impact on the accumulation of these compounds. The clay content cannot explain such observations itself, so we hypothesize there must be other collinear features, such as soil temperature, water holding capacity, and the bioavailability of some elements. In this regard, it is worth noticing that metabolites differently accumulated among soils correlated well with many soil micro- and macronutrients, fostering the hypothesis that soil composition might influence metabolite production at the berry level. This is not trivial since the main message from the current literature is that a soil effect is observed only for unbalanced conditions (Seguin, 1986; Pereira et al., 2005) and that the chemical/mineral uptake provided by the soil does not exert a significant influence on fruit (Van Leeuwen et al., 2004) and wine quality (Poni et al., 2018).

On the other hand, we observed which chemical soil elements are putatively mostly involved in skin metabolite accumulation. Among them, phosphorous seemed to be one of the most relevant elements due to having the highest number of correlations with the secondary metabolites on the berry skin of both cultivars. This element is known for being involved in several physiological processes in the cell (membrane formation, carbohydrate metabolism, protein synthesis, and energy storage and transfer), but there is still little information about its impact on grape traits (Poni et al., 2018). Interestingly, we observed good correlations with total P but not with P-Olsen, suggesting that grapevine is capable of remobilizing the unavailable P even though soil pH is high (retrogradation of phosphorous phenomena), therefore partially dealing with the problem of a low pH-induced availability fraction.

## Conclusions

The present study represents a first-of-its-kind attempt to isolate the soil factor and investigate its effect on plant physiology and fruit quality in terms of metabolite accumulation in berry skin. Similar approaches had already been employed by other authors in the past but always with other plant species, especially monocotyledons, which are mainly herbaceous and easier to transplant and grow.

Our results indicate that soil exerts a relatively limited effect on physiology related to both vegetative and reproductive phase, even though it must be noted that we only focused on a reduced number of parameters and on a few temporal points during plant development. Concerning the skin metabolome, we observed not only a relevant effect of soil factor *per se* but also of its interaction with the genotype and the phenological stage. Amongst the two varieties considered, Corvina and Glera, the red variety Corvina showed higher plasticity, with a higher number of secondary metabolites affected by soil factor and its interactions. Moreover, as a general observation, the ripening stage seems to be more plastic than the complete véraison stage, probably as a consequence of the higher metabolic complexity that characterizes this stage in grape skin. The different behavior of Glera and Corvina varieties grown in different soils was not only quantitative but also qualitative, with different biosynthetic pathways involved. Glera was mainly affected in the biosynthesis of flavonols, whereas Corvina showed a modulation of stilbenoids and their precursors.

Together with statistical analysis aimed at describing the plasticity of secondary metabolism, we also tried to draw correlations between these metabolites and a number of soil physico-chemical parameters. Interestingly, most of the metabolites significantly affected by soil factor in the ANOVA analysis were among those showing the strongest correlations with several physico-chemical characteristics that distinguished the different soils considered, including the composition in terms of micro- and macronutrients, the soil texture, and the percentage of skeleton, giving rise to the hypothesis that soil chemical composition has a direct influence on grape characteristics.

We believe that the approach used in this study could lay the foundations for a whole series of experiments and analyses, including phenology, physiology (partially treated also in the present work), and transcriptomics, aimed at describing and deciphering the concept of terroir from a scientific point of view.

## Data Availability Statement

All datasets generated for this study are included in the article/**Supplementary Material**.

## Author Contributions

CP, AF, and AV performed the research. AV and ML designed the research. CP, AV, and AF helped with specific physiological, metabolic, molecular, and bioinformatics analysis. CP and AV analyzed the data. The manuscript was written by CP, AV, ML, FP, and AF and approved by all other authors.

## Funding

This study was supported by the Starting Grants 2015 CARIPARO project Cod. VANN_START16_01.

## Conflict of Interest

The authors declare that the research was conducted in the absence of any commercial or financial relationships that could be construed as a potential conflict of interest.

## Abbreviations

DOC, Denominazione di Origine Controllata (controlled designation of origin); DOCG, Denominazione di Origine Controllata e Garantita (controlled and guaranteed designation of origin); EC, Electrical Conductance; C.tot, total carbon; C.org, organic carbon; N.tot, total nitrogen; C.N, carbon-to-nitrogen ratio; Al, aluminium; Ca, calcium; Fe, iron; K, potassium; Mg, magnesium; Mn, manganese; Na, sodium; P, phosphorous; S, sulphur; Ti, titanium; As, arsenic; B, baron; Ba, barium; Be, beryllium; Cd, cadmium; Co, cobalt; Cr, chromium; Cu, copper; Li, lithium; Mo, molybdenum; Ni, nickel; Pb, lead; Sn, tin; Sr, strontium; V, vanadium; Zn, zinc; Ca.ex, exchangeable calcium (mg/kg s.s.); K.ex, exchangeable potassium (mg/kg s.s.); Mg.ex, exchangeable magnesium (mg/kg s.s.); Na.ex, exchangeable sodium (mg/kg s.s.); CEC.ex, cation exchangeable capacity (mg/kg s.s.); Ca.mol, exchangeable calcium (cmoli(+)/kg s.s.); K.mol, exchangeable potassium (cmoli(+)/kg s.s.); Mg.mol, exchangeable magnesium (cmoli(+)/kg s.s.); Na.mol, exchangeable sodium (cmoli(+)/kg s.s.); CEC.mol, cation exchangeable capacity (cmoli(+)/kg s.s.); P_Ols, phosphorous Olsen; Cyan-3-glu, Cyanidin-3-O-glucoside; Pet-3-glu, Petunidin-3-O-glucoside; Peo-3-glu, Peonidin-3-O-glucoside; Mal-3-glu, Malvidin-3-O-glucoside; Delph-3-glu, Delphindin-3-O-glucoside; Delph-3-acet, Delphinidin-3-O-(6′′-acetyl-glucoside); Cyan-3-acet, Cyanidin-3-O-(6′′-acetyl-glucoside); Pet-3-acet, Petundin-3-O-(6′′-acetyl-glucoside); Mal-3-acet, Malvindin-3-O-(6′′-acetyl-glucoside); Peo-3-acet, Peonidin-3-O-(6′′-acetyl-glucoside); Delph-3-coum, Delphinidin-3-O-(6′′-p-coumaroyl-glucoside); Cyan-3-coum, Cyanidin-3-O-(6′′-p-coumaroyl-glucoside); Pet-3-coum, Petunidin-3-O-(6′′-p-coumaroyl-glucoside); Peo-3-coum, Peonidin-3-O-(6′′-p-coumaroyl-glucoside); Mal-3-coum, Malvidin-3-O-(6′′-p-coumaroyl-glucoside); Proc, Procyanidin; Myr-3-glr, Myricetin-3-O-glucuronide; Rutin, Quercetin-3-O-rutinoside; Myr- 3-glu, Myricetin-3-O-glucoside; Quer-3-glr, Quercetin-3-O-glucuronide; Quer-3-glu, Quercetin-3-O-glucoside; Kaemp-3-glr, Kaempferol-3-O- glucuronide; Kaemp-3-glu, Kaempferol-3-O-glucoside; Narin-chalc-glu, Naringenin-chalcone-4-O-glucoside; Hex, Hexoside; Resv, Resveratrol.
