# Supplementary material for: The Effect of Soil on the Biochemical Plasticity of Berry Skin in Two Italian Grapevine (V. vinifera L.) Cultivars
Source: Front Plant Sci. 2020 Jun 26;11:822. doi: 10.3389/fpls.2020.00822 (PMC7333541; doi:10.3389/fpls.2020.00822)
Supplement: Supplementary file 7 [file DataSheet_1.pdf]

Supplementary Methods 1: soil physico-chemical analysis

Soil analyses were conducted according to International Union of Soil Science (IUSS) protocols (Violante, 2000). In brief, soil pH and electrical conductivity (EC) were measured potentiometrically using 1:2.5 and 1:5 soil/water extracts, respectively. Calcium carbonate equivalent was determined by the calcimeter method and gravimetric loss of CO2. Particle size analysis was performed according to the hydrometer method, using sodium hexametaphosphate as a dispersant (Gee and Bauder, 1986). Exchangeable bases, including calcium (Ca), magnesium (Mg), potassium (K), and sodium (Na), were extracted using barium chloride (Sumner and Miller, 1996), and their concentrations were determined by inductively coupled plasma-optical emission spectroscopy (ICP-OES) on a SPECTRO CIROS (Spectro Analytical Instruments, Kleve, Germany). Organic carbon was determined by dry combustion in a CNS Vario Macro elemental analyzer (Elementar, Hanau, Germany) and corrected for the inorganic C. Plant available phosphorus (P) was calculated using the Olsen method (Olsen and Sommers, 1982). Olsen extractable P (P_Ols) was obtained by shaking 1.0 g of soil with 20mL of 0.5 mol L−1 sodium bicarbonate solution (pH 8.5) for 30 min. After filtration through 2.5 mm filter paper, P in the extracts was determined by ICP-OES. Macroelements (Al, Ca, Fe, K, Mg, Mn, Na, P, S) were extracted with DTPA solution whereas microelements (B, Ba, Cd, Ch, Cu, Mo, Ni, Pb) by digestion with an HNO_3 plus HCl solution. After filtration through a 0.45 µm syringe filter, elements were determined by ICP-OES.

References

Violante, P. (2000). Metodi di Analisi Chimica del Suolo (Milano: Ministero delle Politiche Agricole e Forestali, Osservatorio Nazionale Pedologico e per la Qualità del suolo, International Union of Soil sciences, Società Italiana Scienza del Suolo).

Gee, G.W., and Bauder, J.W. (1986). Particle-size Analysis. In Methods of Soil Analysis: Part 1—Physical and Mineralogical Methods, (Soil Science Society of America, American Society of Agronomy), pp. 383–411.

Sumner, M.E., and Miller, W.P. (1996). Cation Exchange Capacity and Exchange Coefficients. In Methods of Soil Analysis Part 3—Chemical Methods, (Soil Science Society of America, American Society of Agronomy), pp. 1201–1229.

Olsen, S.R., and Sommers, L.E. (1982). Phosphorus. Methods of soil analyses, part 2. Chem. Microbiol. Prop. Agron. Monogr. 9, 421–422.
