# Supplementary material for: The Effect of Soil on the Biochemical Plasticity of Berry Skin in Two Italian Grapevine (V. vinifera L.) Cultivars
Source: Front Plant Sci. 2020 Jun 26;11:822. doi: 10.3389/fpls.2020.00822 (PMC7333541; doi:10.3389/fpls.2020.00822)
Supplement: Supplementary file 8 [file DataSheet_2.pdf]

Supplementary Methods 2: Biochemical analysis on berry skin

Berry skins were analyzed by LC-MS technology at Sede Boqer campus of Ben-Gurion University of the Negev (Israel). About 200 mg of fine powder per sample was weighted then lyophilized. Metabolites were extracted following a protocol described in Weckwerth et al. (2004) with some adaptation to berry skin as detailed in Degu et al. (2014). Specifically, 1 ml pre-chilled methanol:chloroform:water extraction solution (2.5:1:1 v/v) containing 300 µl of ampicillin (1 mg/ml) in water and 380 µl of corticosterone (1 mg/ml) in methanol as internal standards, was added to each sample. The mixture was briefly vortexed, 100 µl of methanol were added and then placed on a horizontal shaker for 10 min at 1000 rpm. The samples were sonicated for 10 min (Elmasonic S30, Elma, Singen, Germany) and centrifuged for 10 min (14000 rpm, microcentrifuge 5417R, Eppendorf, Hamburg, Germany). The supernatant was decanted into new tubes, mixed with 300 µl of chloroform and 300 µl of Milli-Q water (Millipore, MA, USA), vortexed for 10 s and then centrifuged at 14000 rpm for 5 min. The two resulting phases separated water and methanol (upper phase) from chloroform (bottom phase). The water/methanol phase obtained from the extraction protocol was separated and filtered in vials (0.22µm Millipore, MA, USA) for UPLC analysis.

Samples were run in an Ultra Performance Liquid Chromatography coupled with a Quadrupole Time-of-Flight Mass-Spectrometer (UPLC- QTOF MS, Waters, MA, USA) system operating in either positive and negative ion modes. LC-MS conditions were the same as described in Hochberg et al. (2013) and recently detailed in Reshef et al. (2019). Briefly, metabolites separation was performed by means of a C18 column (ACQUITY UPLC BEH C18 column 100 mm × 2.1 mm, 1.7 µm with ACQUITY UPLC BEH C18 VanGuard Pre-column, 130Å, 1.7 µm, 2.1 mm X 5 mm; Waters MS Technology, Manchester, UK) maintained at 40°C. Leucine enkephalin was used for lock mass calibration. The mobile phase transitioned from 95% water, 5% acetonitrile, 0.1% formic acid (phase A) to 0.1% formic acid in acetonitrile (phase B), with gradient transitioning from 100 to 60% phase A (0–8 min), 60–0% phase A (1 min), a gradual return to 100% phase A (3.5 min), and conditioning at 100 phase A (2.5 min), with a total run time of 15 min.

MassLynxTM software (Waters) version 4.1 was used as the system controlling the UPLC and for data acquisition. The raw data acquired were processed using MarkerLynx application manager (Waters) as described in Hochberg et al. (2013). Metabolites were also annotated based on the consistency of their retention times with those of identified metabolites (Degu et al., 2014) and their fragmentation patterns crossed with the ChemSpider metabolites database (http:// www.chemspider.com/)

References

Degu, A., Hochberg, U., Sikron, N., Venturini, L., Buson, G., Ghan, R., Plaschkes, I., Batushansky, A., Chalifa-Caspi, V., Mattivi, F., et al. (2014). Metabolite and transcript profiling of berry skin during fruit development elucidates differential regulation between Cabernet Sauvignon and Shiraz cultivars at branching points in the polyphenol pathway. BMC Plant Biol. *14*, 188.

Hochberg, U., Degu, A., Toubiana, D., Gendler, T., Nikoloski, Z., Rachmilevitch, S., and Fait, A. (2013). Metabolite profiling and network analysis reveal coordinated changes in grapevine water stress response. BMC Plant Biol. *13*, 184.

Reshef, N., Fait, A., and Agam, N. (2019). Grape berry position affects the diurnal dynamics of its metabolic profile. Plant. Cell Environ. *42*, 1897–1912.

Weckwerth, W., Wenzel, K., and Fiehn, O. (2004). Process for the integrated extraction, identification and quantification of metabolites, proteins and RNA to reveal their co-regulation in biochemical networks. Proteomics *4*, 78–83.
