# Supplementary material for: The Effect of Soil on the Biochemical Plasticity of Berry Skin in Two Italian Grapevine (V. vinifera L.) Cultivars
Source: Front Plant Sci. 2020 Jun 26;11:822. doi: 10.3389/fpls.2020.00822 (PMC7333541; doi:10.3389/fpls.2020.00822)

Figure S1: Principal Component Analysis (PCA) based on physico-chemical characteristics of soils in caissons and those in the sites of origin where they were collected from.

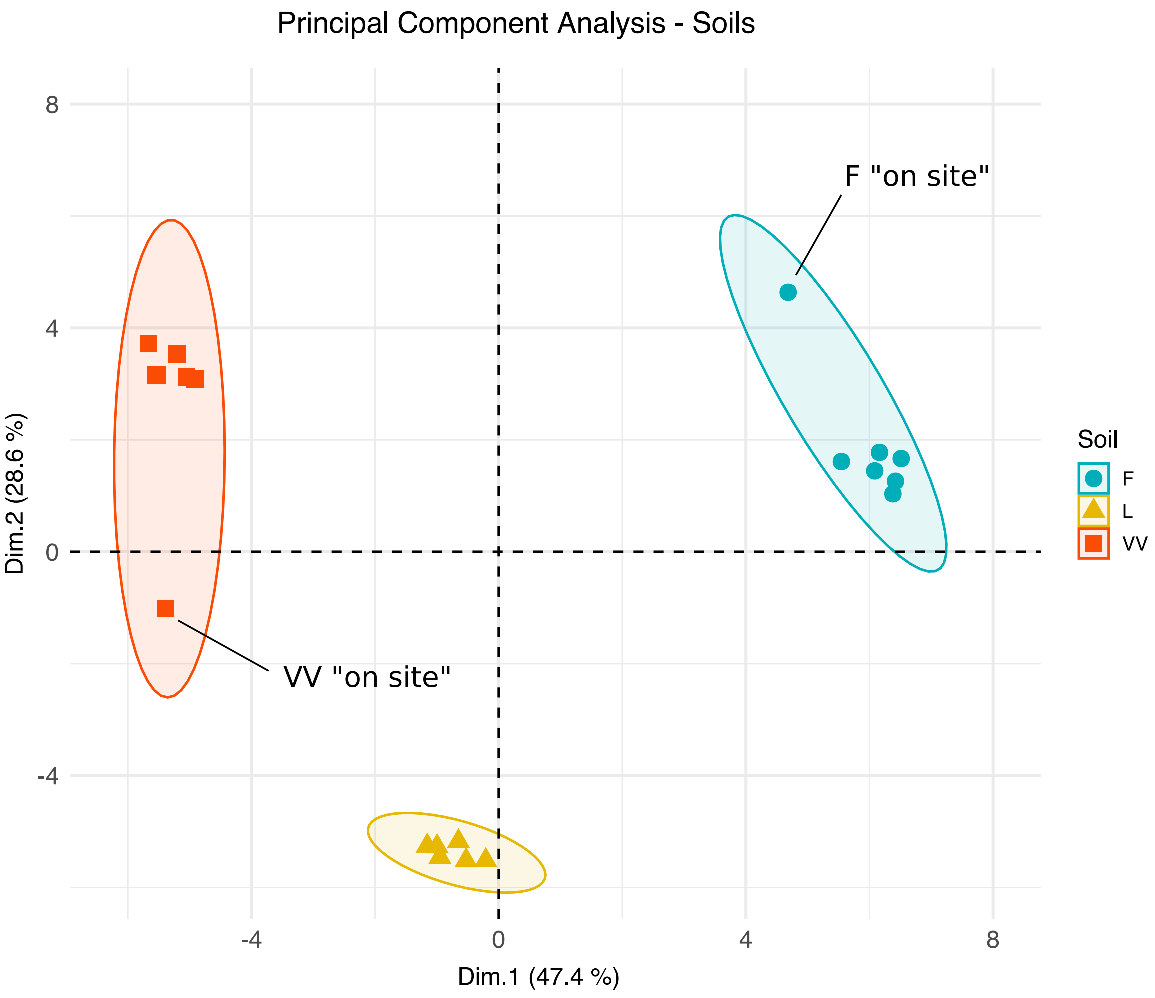

Supplement: Supplementary file 9 [file DataSheet_3.pdf]
